# Evolution of anterior *Hox *regulatory elements among chordates

**DOI:** 10.1186/1471-2148-11-330

**Published:** 2011-11-15

**Authors:** Alfonso Natale, Carrie Sims, Maria L Chiusano, Alessandro Amoroso, Enrico D'Aniello, Laura Fucci, Robb Krumlauf, Margherita Branno, Annamaria Locascio

**Affiliations:** 1 Laboratory of Cellular and Developmental Biology, Stazione Zoologica Anton Dohrn, Villa Comunale, Naples, Italy; 2 Stowers Institute for Medical Research, Kansas City, MO 64110, USA; 3 Department of Soil, Plant, Environmental and Animal Production Sciences, University of Naples "Federico II", Via Università 100, Portici, Italy; 4 Department of Structural and Functional Biology, University of Naples "Federico II", Via Cinthia, Naples, Italy; 5 Department of Anatomy and Cell Biology, Kansas University Medical School, Kansas City, KS 66160, USA; 6 Molecular Cardiovascular Biology, MLC 7020 Cincinnati Children's Hospital Medical Center, 3333 Burnet Avenue, Cincinnati, OH 45229, USA

## Abstract

**Background:**

The *Hox *family of transcription factors has a fundamental role in segmentation pathways and axial patterning of embryonic development and their clustered organization is linked with the regulatory mechanisms governing their coordinated expression along embryonic axes. Among chordates, of particular interest are the *Hox *paralogous genes in groups 1-4 since their expression is coupled to the control of regional identity in the anterior nervous system, where the highest structural diversity is observed.

**Results:**

To investigate the degree of conservation in *cis*-regulatory components that form the basis of *Hox *expression in the anterior nervous system, we have used assays for transcriptional activity in ascidians and vertebrates to compare and contrast regulatory potential. We identified four regulatory sequences located near the *CiHox1, CiHox2 *and *CiHox4 *genes of the ascidian *Ciona intestinalis *which direct neural specific domains of expression. Using functional assays in *Ciona *and vertebrate embryos in combination with sequence analyses of enhancer fragments located in similar positions adjacent to *Hox *paralogy group genes, we compared the activity of these four *Ciona cis*-elements with a series of neural specific enhancers from the amphioxus *Hox1-3 *genes and from mouse *Hox *paralogous groups 1-4.

**Conclusions:**

This analysis revealed that Kreisler and Krox20 dependent enhancers critical in segmental regulation of the hindbrain appear to be specific for the vertebrate lineage. In contrast, neural enhancers that function as *Hox *response elements through the action of Hox/Pbx binding motifs have been conserved during chordate evolution. The functional assays reveal that these *Hox *response *cis*-elements are recognized by the regulatory components of different and extant species. Together, our results indicate that during chordate evolution, *cis*-elements dependent upon Hox/Pbx regulatory complexes, are responsible for key aspects of segmental *Hox *expression in neural tissue and appeared with urochordates after cephalochordate divergence.

## Background

In all the animal species from insects to vertebrates *Hox *genes play a key role in determining anterio-posterior (AP) identities. The clustered organization and spatio-temporal colinearity of expression of *Hox *genes in many species are believed to be important for their functional roles [1]. The availability of genomic sequences from an increasingly large number of species has shed light on many aspects of the evolution of *Hox *gene organization. However, this data has also opened new questions on the origin of *Hox *genes and on the mechanisms that controlled their evolution [2,3]. A single set of *Hox *genes is present in all invertebrates and non-vertebrate chordates analysed so far. Two rounds (2N) of genome-wide duplication led to the formation of four *Hox *clusters seen in most vertebrates, while an additional (3N) round of duplication and divergence in ray finned fishes has led to seven or eight *Hox *clusters [3].

Recent genomic analyses have demonstrated that only vertebrates have a compact and well organized *Hox *cluster, while most of the other chordates and invertebrates analysed so far have "an intact but disorganized, a split or an atomized cluster" with a highly variable gene number [2]. Echinoderms have a single *Hox *cluster that has undergone significant rearrangements of the member gene positions. The sequencing of the genome of *Strongylocentrotus purpuratus *revealed a large *Hox *cluster with eleven genes that have undergone rearrangements of transcriptional orientation and gene order. The *Hox5 *gene is the most 3' gene and the *Hox1-3 *lie near the members of the cluster expressed in posterior regions [4]. The cephalochordate amphioxus has a single and intact cluster quite similar to compact vertebrate organization and the *Hox1, 2, 3, 4 *and *6 *genes show both temporal and spatial colinearity of expression along the anteroposterior axis of the developing neural tube [5,6]. Nevertheless, there has been a further expansion of the 5' genes generating a total of fifteen paralogous groups [7-9]. Urochordates have nine *Hox *genes and both the larvacean *Oikopleura dioica *and the ascidian *Ciona intestinalis *species have lost *Hox *genes of the central paralogous groups [10-12]. It has been suggested that rapid embryogenesis and a simplification in body organization are at the basis of the breakdown of central *Hox *genes in these organisms.

*Oikopleura *has completely lost the clustered organization of *Hox *genes while still maintains some anteroposterior colinearity in the notochord, neural tube, tail muscle and epidermis [12]. In *Ciona*, only some of the nine *Hox *genes correspond to the *Oikopleura *complements and they are located on two chromosomes and interspersed with many other unrelated genes. There is no evidence for temporal colinearity, however spatial colinearity is partially maintained [13]. In particular, *CiHox1, CiHox3, CiHox10, CiHox5 *and *CiHox12 *exhibit a spatially coordinated and restricted pattern of expression along AP axis of the central nervous system at the level of the visceral ganglion and nerve cord, which are considered homologous to vertebrate hindbrain and spinal cord respectively [13-16]. The other *Ciona Hox *genes (*CiHox2, CiHox4 *and *CiHox13*) appear to have lost their function in development of the nervous system and are associated the roles in other tissues such as mesenchyme. The *Ciona Hox2 *gene, unlike its vertebrate counterparts, has lost any evidence of spatio-temporal colinearity and is expressed only at the larva stage, in trunk lateral cells [13].

Both ascidians and amphioxus lack a segmented hindbrain but the restricted expression patterns of anterior *Hox *genes and of other molecular markers show clear homologies with vertebrates in nervous system patterning [6,13-15]. Vertebrate *Hox *genes exert a fundamental role in hindbrain formation and segmentation [17,18]. They show sharp and nested anterior limits in the rhombencephalon and each *Hox *gene has a specific and different expression profile [19]. These expression profiles are temporally and spatially defined through the combined action of different regulatory elements. Regulatory studies in the vertebrate hindbrain have demonstrated that different combinations of *cis*-elements and regulatory components are used to establish and maintain segmental *Hox *expression domains during development.

Trying to understand which of the *cis*-regulatory modules that control and modulate *Hox *genes expression may be common to all chordates and which ones appeared only in specific lineages represents an interesting challenge. Gene duplication and divergence provides an opportunity to acquire new genetic material that during evolution will permit diversification and appearance of new structures.

All chordates are characterized by the same body plan with a dorsal hollow neural tube, a notochord and lateral muscles but the very rudimentary cephalic structures typical of protochordates evolved and led to the appearance of a considerable number of innovations in vertebrates. Vertebrates unlike the other chordates are characterized by the neural crest, placodes and a complex brain [20].

Hox genes played a key role in the morphological evolution of these structures. They control the correct segmentation patterning of these structures and define their antero-posterior identity. From an evolutionary point of view, the duplication and subsequent divergence of *Hox *genes are believed to have contributed to the formation of the vertebrate innovations. In particular, changes in gene regulatory regions are considered a driving force for the evolution of more complex body plan structures [9].

Studies by phylogenetic footprinting have identified conserved noncoding regions but often large phylogenetic distances make it difficult to establish the real significance of the results obtained. The sequencing of the *Hox *cluster of the european amphioxus *Branchiostoma lanceolatum *and its comparison with the other clusters of the amphioxus *Branchiostoma floridae *and vertebrates permitted, at least in part, to overcome the phylogenetic distances and to identify several putative regulatory regions [8,21]. Nonetheless, further characterization of these sequences will be necessary to establish their biological significance. Amphioxus *Hox1-3 *regulatory elements tested in mouse and chicken embryos revealed the presence of conserved neural elements adjacent to the *AmphiHox1 *and *AmphiHox3 *genes dependent upon retinoic signalling for activity [22,23]. These control regions contained retinoic acid response elements (RAREs) of the DR5-type located at the 3' end of *AmphiHox1 *and at the 5' end of *AmphiHox3 *which are responsible for expression of these genes in vertebrate neural crest and neural tube from rhombomere 6 to posterior without any specific segmentation [22,23]. This is consistent with data showing that the endogenous *AmphiHox1 *and *AmphiHox3 *genes respond to retinoic acid [5,24,25]. Retinoic acid excess directly induces altered expression of these genes in gastrula embryos [25]. Furthermore, these results also demonstrated that *AmphiHox1*, like its vertebrate counterparts *Hoxa1 *and *Hoxb1*, is a direct target of retinoid signalling in the nervous system [5,24]. Among chordates, the sensitivity to retinoic acid seems to be less conserved in urochordates. The larvacean *Oikopleura *lacks genes of the retinoic acid pathway and does not show any homeotic posteriorization after RA treatment [26]. In the ascidian *Ciona intestinalis*, only *CiHox1 *clearly responds to exogenous retinoic acid and it has an RA responsive element for its epidermal expression but it seems to lack RAREs at its 3' end controlling neural expression [22,27-29].

Regulatory and mutational analyses in mice and other vertebrate species have shown that Krox20 and Kreisler transcription factors play crucial roles in the process of hindbrain segmentation by regulating rhombomere (r)-specific expression of the *Hoxa2, Hoxa3, Hoxb2 *and *Hoxb3 *genes [17,30-33]. However, regulatory analyses of the *AmphiHox1-3 *loci suggests that Krox20 and Kreisler dependent neural elements have not been conserved in amphioxus [23]. Previous studies on the *Ciona CiHox3 *gene and its neural specific regulatory element(s) revealed a similar divergence of Krox20 and Kreisler-dependent *Hox3 *control elements between vertebrates and ascidians, but showed that a certain degree of conservation exists with respect to *Hox *response elements capable of mediating auto- or cross-regulatory inputs from *Hox *genes [15]. This is interesting because in vertebrates, following initial activation by transient inputs from retinoids, Krox20 and/or Kreisler, the segmental expression of *Hoxb1, Hoxb2, Hoxa2 *and *Hoxa3 *are maintained through separate *cis*-modules by positive auto- and cross-regulatory loops, that involve interactions between Hox proteins and the Meis/Prep and Pbx co-factors. For example, the murine *Hoxa3 *and *Hoxb3 *genes are first activated in r5 and r6 under the control of an enhancer with conserved Kreisler binding sites [31,32], while in later stages only *Hoxa3 *is maintained in r5 through an auto-regulatory loop dependent upon conserved Hox/PBC sites [31]. Similarly, following activation of *Hoxa1 *and *Hoxb1 *by retinoids, the expression of *Hoxb1, Hoxb2 *and *Hoxa2 *in r4 is achieved by a series of auto- and cross-regulatory enhancers under the control of dimeric and trimeric Hox/Pbx/Prep complexes [34-37]. These studies illustrate that auto-and cross-regulatory inputs from *Hox *genes themselves are an important component of *Hox *regulation.

To investigate how cis-regulation of *Hox *genes changed and evolved in the chordate lineage, we have used regulatory analyses in *Ciona *and vertebrates to analyse and compare various *Hox *regulatory regions of three chordate species, amphioxus, ascidian and mouse. In particular, we focused our attention on the elements controlling anterior *Hox *genes expression in the nervous system because the CNS is the territory where major structural differences can be observed in these chordate organisms and it is also the only one where anterior *Hox *genes of all three species are expressed during embryonic development. We identified several new control regions in *Ciona *and our results indicate that during chordate evolution, *cis*-elements dependent upon Hox/Pbx regulatory complexes, are responsible for key aspects of segmental *Hox *expression in neural tissue and appeared with urochordate after cephalochordate divergence. However, segmental regulation in the vertebrate hindbrain mediated by factors such as Kreisler and Krox20 appears to be specific for the vertebrate lineage.

## Results

**Figure 1:**
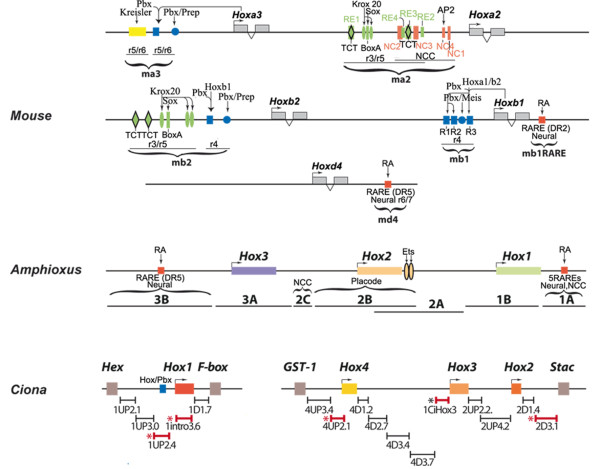
**Genomic organization of the mouse, amphioxus and *Ciona *anterior *Hox *regulatory elements assayed by transgenesis in *Ciona *or vertebrate embryos**. *Ciona *positive regulatory fragments are evidenced by a red asterisk while the black asterisk indicates location of *CiHox3 *enhancer.

To enable evolutionary comparisons to probe the degree of conservation of *Hox *regulatory elements directing anterior neural expression in mouse, amphioxus and *Ciona *we selected a series of *Hox *rhombomere-specific enhancer elements characterized in mouse embryos for their ability to specifically direct segmental expression of *Hox *genes in paralogous groups 1, 2, 3 or 4. In particular, we examined two *Hoxb1*, a *Hoxb2*, a *Hoxa2*, a *Hoxa3 *and a *Hoxd4 *enhancer. Each enhancer contains a different set of the Krox20, Kreisler, Pbx/Meis and RARE binding sites, whose characteristics are indicated in Figure 1[31,35,37-40].

All the *Hox1, Hox2 *and *Hox3 *amphioxus genomic fragments tested by Manzanares et al. [23] in mouse and chicken embryos have been chosen as representative of anterior *Hox *genes regulatory elements of cephalochordates (Figure 1).

We then needed to map and identify functional elements in *Ciona*. Towards this end we performed transcriptional assays to scan for fragments in and around *Hox *genes in paralogy groups 1-4 capable of directing reporter activity in *Ciona *embryos. We identified four *cis*-elements as described below (marked by red *, Figure 1).

### *CiHox1 *regulatory elements

**Figure 2:**
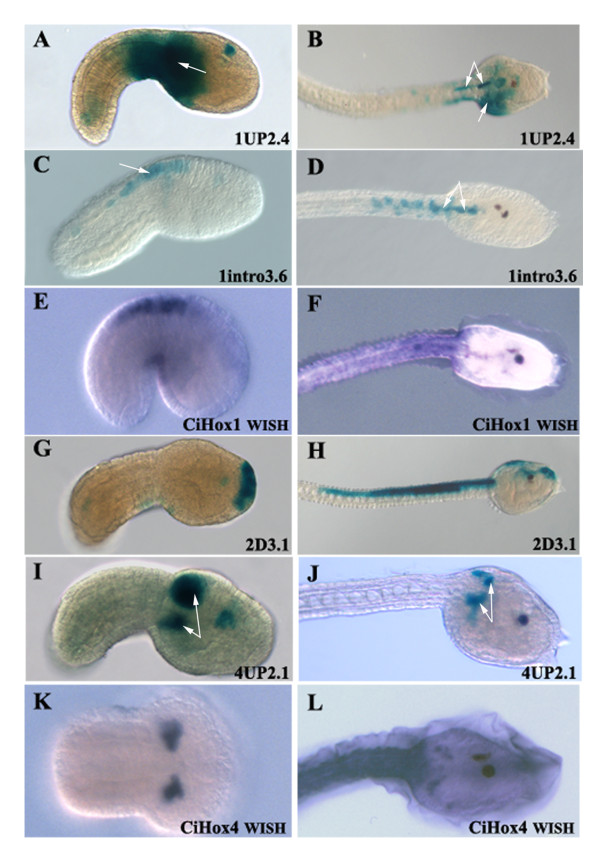
**Expression territories of *CiHox1, CiHox2 *and *CiHox4 *positive cis-elements**. A-D) LacZ expression of constructs 1UP2.4 and 1intro3.6 in electroporated embryos at tailbud and larva stages. E, F) *CiHox1 *endogenous expression profile by whole mount in situ hybridization at the same embryonic stages. LacZ and endogenous *CiHox1 *are both expressed in the epidermis and CNS between the trunk and the tail at tailbud (A, C, E) and larva (B, D, F) stages. Construct 1UP2.4 shows also ectopic expression in the sensory vesicle (A, B). G, H) Reporter gene expression of construct 2D3.1 in the future palps at tailbud stage (G) and in the palps, sensory vesicle at larva stage (H). I, J) LacZ expression of construct 4UP2.1 in the anterior CNS and mesenchymal pockets at tailbud (I) and only in the mesenchymal pockets at larva stage (J). K, L) whole mount in situ hybridization of *CiHox4 *gene in the mesenchymal pockets of embryos at the same developmental stages. White arrows indicate LacZ expression corresponding to the endogenous gene expression. Anterior is on the right and posterior on the left. All the embryos are on a dorsal view, except A, C and E that are on a lateral view.

**Figure 3:**
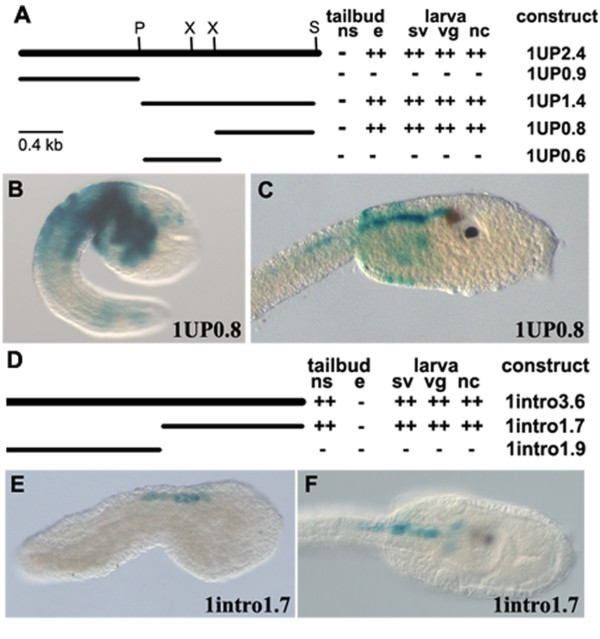
**Summary of 1UP2.4 and 1intro3.6 deletion constructs and of their activity in transgenic *Ciona *embryos**. A, D) On the left schematic representation of 1UP2.4 (A) or 1intro3.6 (D) constructs and their deleted constructs. The restriction sites used for the preparation of the transgenes are also indicated (P, PstI; S, *Sma*I; X, *Xba*I). Right side, constructs names and the tissues where the reporter gene is expressed. The number of crosses is indicative of days of staining. B, C) Expression of 1UP0.8 construct in the same territories of the endogenous *CiHox1 *gene in the epidermis of tailbud (B) and epidermis and CNS of larva embryos (C). E, F) Nervous specific expression of 1intro1.7 construct at tailbud (E) and larva (F) stages corresponding to endogenous neural expression of *CiHox1*. Anterior is on the right; B, C, E, lateral view; F, dorsal view.

A genomic sequence of about 20 kb encompassing *CiHox1 *and the two non-*Hox *adjacent genes (*Hex *and *Fox*) was obtained from the JGI genome sequencing project of *Ciona intestinalis *[41] and was analysed by the Nix programme of the UK Human Genome mapping project (HGMP). The resulting *CiHox1 *gene structure was used to generate a series of five genomic fragments extending through the intergenic region downstream of *Hex *through *CiHox1 *to the start of *Fox *adjacent genes (Figure 1). The fragments were cloned into a reporter vector containing *LacZ *as reporter gene and *CiHox3 0.2 *as the basal promoter [15]. These constructs were electroporated into *Ciona *embryos to identify putative *CiHox1 *enhancer elements with regulatory potential by staining for *LacZ *reporter gene expression in embryos at tailbud and larva stages. The genomic regions *1UP2.1, 1UP3.0 *and *1D1.7 *did not give any specific *LacZ *activation (data not shown). In contrast, fragments *1UP2.4 *and *1intro3*.6 appear to recapitulate the expression profiles of the endogenous *CiHox1 *gene (Figures 2A-F). In particular, endogenous *CiHox1 *is expressed in the epidermis and CNS at the junction between the trunk and the tail at tailbud stage (Figure 2E) and in the corresponding epidermis, visceral ganglion and anterior caudal neural tube at larva stage (Figure 2F). The construct *1UP2.4*, extending from position -2924 to -573 at the 5' end of the gene, activates *LacZ *expression in the epidermis at the junction between the trunk and the tail at both tailbud and larva stages (Figures 2A, B) and recapitulates *CiHox1 *endogenous epidermal expression (Figures E, F). This construct also recapitulates *CiHox1 *expression in the corresponding CNS, at level of the visceral ganglion and anterior caudal neural tube, but only at larva stage (Figure 2B). There is a specific but ectopic domain of *LacZ *expression in the most anterior part of the CNS that can be observed at tailbud stage (Figure 2A) and in the sensory vesicle at larva stage (Figure 2B).The construct *1intro3.6*, containing a genomic fragment encompassing the second intron of *CiHox1 *is responsible for early and late activation in the CNS, recapitulating endogenous *CiHox1 *neural expression at both tailbud and larva stages. In particular, its expression was observed in the dorsal nerve cord at the junction between the trunk and the tail at tailbud stage (Figure 2C) and in the visceral ganglion and anterior neural tube at larva stage (Figure 2D). At this stage, an ectopic signal was also visible in the sensory vesicle. The analysis of a series of progressively deleted fragments of these two constructs (Figures 3A, D), identified a 0.8 kb DNA fragment (*1UP0.8*) able to activate reporter gene expression in the same territories of *1UP2.4 *construct and specifically, in the epidermis of tailbud and larva embryos and in the CNS of larva embryos (Figures 3B, C). The second fragment of 1.7 kb (*1intro1.7*) reproduces *1intro3.6 *construct and thus nervous specific *CiHox1 *expression at the junction between the trunk and the tail at both tailbud and larva stages (Figures 3E, F). These results clearly indicate that the *1UP0.8 *fragment, located 5' of the *CiHox1 *gene, contains the regulatory elements controlling its expression in the epidermis at tailbud and larva stages and in the CNS at level of visceral ganglion and anterior nerve cord only at larva stage. The genomic region of 1.7 kb located in the second intron of *CiHox1 *(*1intro1.7*) is responsible for both early and late activation of the *CiHox1 *gene in the nervous system.

### *CiHox2 *and *CiHox4 *regulatory elements

A genomic region encompassing the *CiHox2, CiHox3 *and *CiHox4 *genes was also analysed by using the same enhancer scanning strategy and reporter vectors adopted above for *CiHox1*. The ten fragments analysed lie between two adjacent non-*Hox *genes, and extend from the end of *GST-1*, located 5' of *CiHox4*, to the start of *Stac*, located 3' of *CiHox2 *(Figure 1). Previous analyses from our group examined the regions surrounding *CiHox3 *and identified an 80 bp element located at the 5' end of *CiHox3 *(indicated by an asterisk in Figure 1). This element is capable of mediating neural specific reporter expression [15] at level of the sensory vesicle and visceral ganglion (Table 1) and this last signal reproduces endogenous *CiHox3 *expression.

**Table 1:** Neural territories of expression of positive *Ciona*, amphioxus and mouse *Hox *cis-elements

Name	Species	Expression in *Ciona *					Expression in Vertebrate
1UP1.4	*C. intestinalis*	SV	VG	NC	**-**	**-**	r4

1intro1.7	*C. intestinalis*	SV	VG	NC	**-**	**-**	hindbrain

2D0.8	*C. intestinalis*	SV	VG	**-**	Ph	TSN	**-**

1CiHox3	*C. intestinalis*	SV	VG	**-**	**-**	**-**	r4 r6/NC

4UP1.3	*C. intestinalis*	SV	**-**	**-**	**-**	**-**	**-**

2B	*B. floridae*	SV	VG	**-**	Ph	TSN	V,VIII ganglia

mb1	*M. musculus*	SV	**-**	NC	**-**	**-**	r4

ma3	*M. musculus*	**-**	**-**	**-**	**-**	TSN	r5/r6 NC

Among the four genomic fragments of *CiHox2 *tested, only construct *2D3.1*, located 3' of the gene, is able to activate reporter gene expression in the future palps of the embryo at tailbud stage (Figure 2G) and in the palps and CNS, at level of the sensory vesicle, at the larva stage (Figure 2H). This construct also activates reporter expression in tail muscles, but this pattern of staining is highly variable among the embryos and has been considered not specific. The expression profile of construct *2D3.1 *does not recapitulate that of the endogenous *CiHox2 *gene, which is expressed only at larva stage in the trunk lateral cells [13]. This suggests that additional fragments may work to restrict its activity or that these *cis*-elements located 3' of *CiHox2 *might function on the adjacent non-*Hox Stac *gene. To probe this latter possibility, we performed whole mount in situ hybridization experiments on embryos at tailbud and larva stages in order to establish *Stac *gene expression profiles. *Stac *is expressed in the epidermis at larva stage and is not expressed at tailbud stage (data not shown). Thus, *Stac *expression is completely different from that of *CiHox2 *gene and the pattern of reporter staining mediated by construct *2D3.1*.

**Figure 4:**
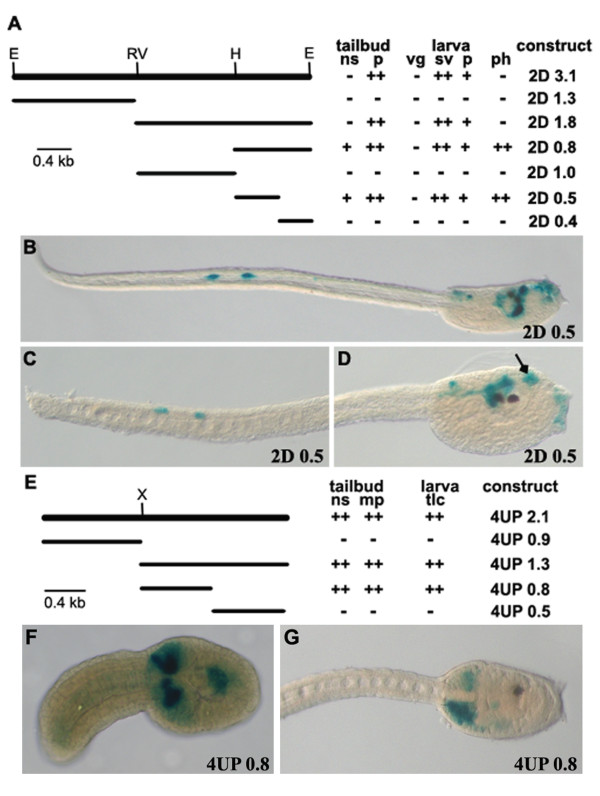
**Cis-regulatory activity of the 2D3.1 and 4UP2.1 deletion constructs**. A) Schematic representation of 2D3.1 deleted fragments and of their expression in *Ciona *embryos at tailbud and larva stages. B) Construct 2D0.5 is active in the palps and sensory vesicle, as for construct 2D3.1, and also in the caudal sensory neurons (C), in the pharynx and visceral ganglion (D). E) Summary of 4UP2.1 deletion constructs and of their activity in transgenic *Ciona *embryos at tailbud and larva stages. F, G) 4UP 0.8 construct is expressed in the anterior CNS at tailbud stage (F) and in the mesenchymal pockets at both tailbud and larva stages (F, G). E, *EcoR*I; H, *Hind*III; RV, *EcoR*V; X, *Xba*I.

To further investigate the regulatory potential of region *2D3.1*, to specifically activate gene expression in the *Ciona *CNS we generated a deletion series (Figure 4A) and scored for reporter activity (Figures 4B-D). This analysis led to the identification of a 0.5 kb DNA fragment (*2D0.5*) which not only reproduces the expression profile of fragment *2D3.1 *in the sensory vesicle and the palps, but also activates *LacZ *expression in the pharynx, the visceral ganglion (Figure 4D) and tail sensory neurons (TSN) (Figure 4C, Table 1). These results suggest that *2D3.1 *contains an enhancer region capable of activating expression in neural and other tissues in combination with a repressor element that partially blocks its activity. Other repressor regions might reside in flanking regions of *2D3.1 *and serve to further restrict the potential of the enhancer in generating the endogenous pattern of *CiHox2 *expression.

To screen for the regulatory element(s) responsible for *CiHox4 *expression we tested six fragments, two located at the 5' end of the gene and four located at the 3' end (Figure 1). Of these regions, only *4UP2.1*, located just upstream of the *CiHox4 *coding sequence, is able to activate *LacZ *expression. We observed staining specifically in the mesenchymal pockets (Figures 2I, J) which reproduces the *CiHox4 *expression profile at tailbud and larva stages, as revealed by in situ hybridization experiments (Figures 2K, L). Furthermore, this construct is also able to activate expression in the most anterior central nervous system at tailbud stage (Figure 2I). Deletion analysis of the sequence contained in construct *4UP2.1 *has identified a 0.8 kb region (*4UP0.8*) responsible for this regulatory activity (Figure 4E). This *4UP0.8 *fragment reproduces the expression profile of construct *4UP2*.1 both in the anterior CNS and in the mesenchymal pockets at tailbud stage (Figure 4F) and only in the mesenchymal pockets at larva stage (Figure 4G).

### Amphioxus anterior *Hox *regulatory elements in *Ciona*

To begin our functional comparisons between *Ciona*, amphioxus and mouse, we first tested by electroporation in *Ciona *embryos all the amphioxus genomic fragments tested by Manzanares et al. [23] in mouse and chicken embryos (Figure 1). We prepared a series of constructs containing the amphioxus *1A, 1B, 1C, 2A, 2B, 2C, 3A, 3B and 3C *genomic elements, together with *LacZ *reporter vectors using either human *β-globin *or *Ciona CiHox3 0.2 *[15] as basal promoters. These two basal promoters work both in *Ciona *embryos with the difference that the human *β-globin *is more efficient in gene activation but gives also more non-specific staining in the mesenchyme in comparison to the *CiHox3 0.2 *element (personal unpublished information).

**Figure 5:**
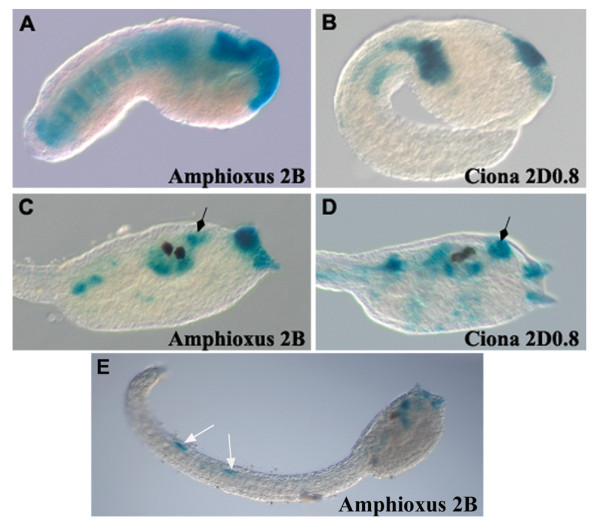
**Analysis of the amphioxus 2B regulatory element in *Ciona *embryos**. Expression mediated by amphioxus 2B element in anterior nervous system, pharynx (black arrow) and palps in tailbud (A) and larva (C) embryos is very similar to *Ciona *construct 2D0.8 expression at the same developmental stages (B, D). Amphioxus 2B is also active in notochord cells at tailbud stage (B) and in the tail sensory neurons at larva stage (E, white arrows).

The majority of the amphioxus control regions, which function in mouse and chick embryos [23], do not display regulatory activity in *Ciona*. The only amphioxus element that gave a positive result in *Ciona *is construct *2B*. This construct contains a 5.6 kb amphioxus genomic region encompassing the 5' end of *Hox2*, its coding region and part of its 3' end (Figure 1). As also reported by Wada et al. [42], this element leads at tailbud stage to the activation of *LacZ *in the most anterior part of the embryo in the precursors of the palps, sensory vesicle and pharynx (Figure 5A). We also observed expression in the notochord cells not previously reported. At larva stage construct *2B *is specifically active in the corresponding anterior territories observed at tailbud stage (Figure 5C). We also observed in a high percentage of electroporated larvae a clear expression in the tail sensory neurons (Figure 5E).

This expression profile of the amphioxus *2B *element is very similar to that obtained with the *Ciona 2D0.8 *construct (Figures 5B, D). Constructs *2D0.8 *and *2B *at tailbud stage have similar activities in the palps precursors and anterior CNS (Figures 5A, B) and, at larva stage, both fragments are able to activate reporter gene expression in the palps, the pharynx and the sensory vesicle (Figures 5C, D). The amphioxus *2B *fragment contains two Ets binding sites located at the 3' end of the amphioxus *Hox2 *gene that are responsible for its expression in these territories [42]. It is interesting to note that the *Ciona 2D0.8 *fragment is located at the 3' end of the *Hox2 *gene, suggesting that these regulatory elements may be related and already present in the common ancestor to cephalochordates and urochordates. For sequence comparison of these two fragments see below the paragraph of "in silico analyses".

### Mouse *Hox *enhancers in *Ciona*

**Figure 6:**
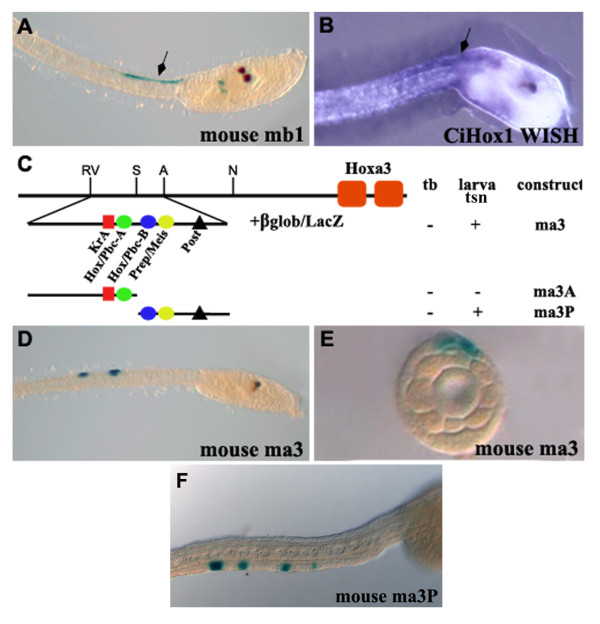
**Analysis of the mouse mb1 and ma3 regulatory elements in *Ciona *embryos**. A) Lateral view of a transgenic *Ciona *embryo at larva stage showing mb1 expression in the sensory vesicle and anterior nerve cord. B) This latter territory (arrow) corresponds to that of the endogenous *CiHox1 *gene. C) Schematic representation of mouse ma3 deleted fragments and their corresponding activity. D) Lateral view of a transgenic larva electroporated with construct ma3, showing expression in a couple of tail sensory neurons. E) Transverse gelatin section at level of the tail sensory neuron marked by ma3. F) The mouse ma3P construct shows the same expression of the ma3 construct in the tail sensory neurons.

Next we tested in *Ciona *embryos a series of *Hox *rhombomere-specific enhancer elements characterized in mouse embryos for their ability to specifically direct segmental expression of *Hox *genes of paralogous groups 1, 2, 3 or 4 and, as shown in Figure 1, containing a different set of the Krox20, Kreisler, Pbx/Meis and RARE binding sites [31,35,37-40]. Among these elements only two of them were capable of mediating reporter staining and only at the larva stage (Table 1). One, *mb1 *construct, is a 650 bp mouse *Hoxb1 *enhancer, located at the 5' end of the gene (Figure 1), which serves as a *Hox *response element through three cooperating repeats recognised by the Hox/Pbx complexes. In vertebrates, this *Hoxb1 *enhancer activates expression specifically in rhombomere 4 and its associated neural crest cells [35]. In *Ciona *embryos, construct *mb1 *activates *LacZ *expression at larva stage in the posterior part of the sensory vesicle and in the most anterior part of the caudal neural tube (Figure 6A). This expression profile is very similar to that observed with the endogenous *CiHox1 *gene (Figure 6B) indicating that the mouse element recapitulates the major part of endogenous *CiHox1 *expression in the nervous system.

The construct *ma3 *is also active in *Ciona *larvae (Table 1). It contains an enhancer located 5' of the mouse *Hoxa3 *gene with five binding motifs for Kreisler, Hox/Pbx and Prep/Meis complexes (Figure 6C) [31]. In *Ciona *embryos, the mouse *Hoxa3 *enhancer, activates reporter gene expression at larva stage in the peripheral nervous system and specifically in the tail sensory neurons (Figure 6D). A transversal section of an electroporated larva, clearly shows the position of the stained cell on the surface of the tail (Figure 6E). To determine which of the motifs are important for this activity, the ma*3 *regulatory sequence, has been subdivided into two smaller fragments (Figure 6C). The region *ma3A *contains the KreislerA site and a single Hox/Pbc-A motif, while the *ma3P *region contains the adjacent Hox/Pbc-B motif, the Prep/Meis and the posterior elements. Positive results have been obtained only following electroporation of the *ma3P *construct and it reproduces the results obtained with *ma3 *in the tail sensory neurons (Figure 6F). Thus, the Kreisler motif is not necessary for activity and the posterior element is the most probable candidate to explain this expression in caudal sensory neurons. Even though this profile does not recapitulate any typical *Hox *expression, it is to note that also the *Ciona 2D0.5 *and amphioxus *2B *fragments are expressed in this territory. Again this result indicates the presence of conserved elements among the *Hox *genes of chordates. Looking for common binding sites, an in silico analysis of these sequences has been done, see below.

### *Ciona Hox *regulatory elements in chicken

**Figure 7:**
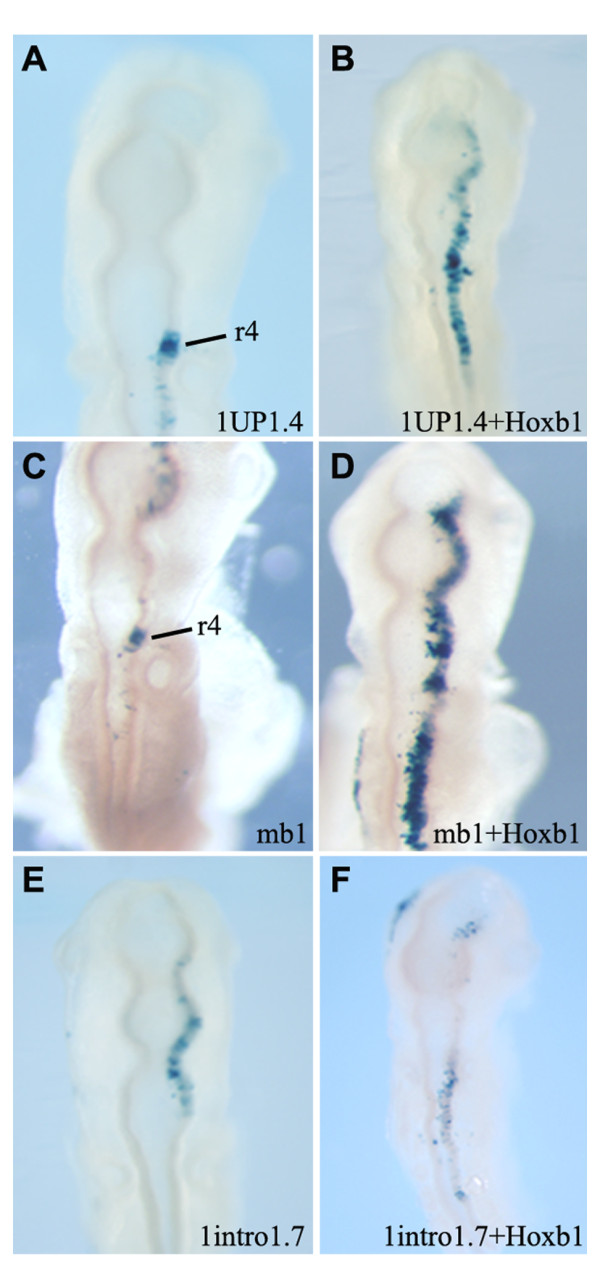
**The activity of *Ciona CiHox1 *cis-elements in transgenic chicken embryos**. A-B) Dorsal view of chicken embryos electroporated with 1UP1.4 construct with 1UP1.4 plus Hoxb1 expression constructs. 1UP1.4 activity is restricted to rhombomere 4 (A) and is expanded by Hoxb1 overexpression (B). C) As control, the mouse mb1 element is expressed in r4. D) Hoxb1 overexpression induces mb1 expanded activation in the whole hindbrain. E, F) Chicken embryos showing 1intro1.7 construct expression in the rhomboencephalon. F) 1intro1.7 activity is not influenced by Hoxb1 overexpression.

To test whether the *Ciona *regulatory elements could function with vertebrate transcriptional machinery, the genomic fragments active in *Ciona *CNS and contained in the *1UP1.4, 1intro1.7, 2D0.8 *and *4UP1.3 *constructs were tested by electroporation in developing chicken embryos. A series of experiments using embryos at different stages of development revealed that constructs *2D0.8 *and *4UP1.3 *were not functionally active while *1UP1.4 *and *1intro1.7 *were able to direct *LacZ *reporter expression in chicken CNS. In particular, the *1UP1.4 *fragment directed positive *LacZ *expression specifically in rhombomere 4 in chicken embryos at HH stage 8-10 (Figure 7A). This is interesting because enhancers from the mouse *Hoxb1, Hoxb2 *and *Hoxa2 *genes which are highly conserved in vertebrates and capable of directing reporter expression in an r4-restricted manner have been shown to serve as *Hox *response elements dependent upon the binding of Hox/Pbx and Meis complexes. Sequence analysis of the *1UP1.4 *region indicated the presence of multiple Hox/Pbx and Meis consensus binding sites suggesting that it too might function as a *Hox *response element (see below the "in silico analyses" paragraph). Therefore, we tested the ability of the *1UP1.4 *region to respond to ectopic expression of *Hoxb1*. As a positive control, in parallel we performed a similar trans-activation experiment using the mouse r4 enhancer region mb1, which was previously shown to contain Hoxb1/Pbx/meis elements responsible for directing expression in r4 (Figure 7C and [35]). We found that ectopic expression of *Hoxb1 *is able to expand reporter expression mediated by both *1UP1.4 *and *mb1 *throughout the entire hindbrain (Figure 7B). This result indicates that the *1UP1.4 *region contains a conserved Hox/Pbx regulatory element that mediates a response to group 1 Hox proteins.

The *1intro1.7 *region was the other *Ciona *regulatory element active in the chicken hindbrain, but domains of expression were not rhombomere specific. As shown in Figure 7E, it is expressed in a variable manner along the whole rhomboencephalon but for the purpose of comparison with the *1UP1.4 *region we tested its ability to mediate a *Hox *response. Ectopic expression of *Hoxb1 *did not induce changes in its patterns of reporter expression mediated by the *1intro1.7 *region (Figure 7F). This suggests that the *1intro1.7 *region contains an unknown but conserved specific regulatory potential which is not Hox/Pbx dependent and thus different from that of the *1UP1.4 *DNA fragment. It could be a neural RARE element that Kanda et al. (2009) hypothesized be present in this genomic region [29].

### In silico analyses of *Ciona*, amphioxus and mouse regulatory sequences

By using the *Ciona*, amphioxus and mouse regulatory sequences, we performed an in silico analysis to identify similarity with potential binding sites for known transcription factors included in the Transfac Professional Database 11.4 [43]. In this analysis we considered the sequences listed in Table 1. In particular, we considered the *Ciona 1UP1.4, 2D0.8 *and *4UP1.3 *and the mouse *mb1 *and *ma3 *sequences tested in this work, we also included the mouse *mb2, ma2 *and *mb1*RARE that did not show any expression in *Ciona *embryos and the *CiHox3 *fragment of 2.3 kb (*1CiHox3*), studied by Locascio et al. [15] and active in *Ciona *sensory vesicle and visceral ganglion. We first determined the potential binding sites for different classes of transcription factors present in each sequence identifying similarity to varied consensus patterns belonging to each class. Then we identified those consensus sequences belonging to common classes of TF binding sites using an "in house" program to process the results obtained by the similarity search approach [32]. In particular, we grouped these sequences on the basis of common expression profiles in *Ciona *or vertebrate embryos (Table 1). We compared all the sequences that are active in *Ciona *sensory vesicle (*1UP1.4, 2D0.8, 1CiHox3, 4UP1.3 *and the mouse *mb1*). The binding sites that are common to all of them have been extracted (see Additional File 1: Table S1). We, then, analyzed the other mouse regulatory sequences not active in the sensory vesicle for the presence of the same binding sites. As shown in Table 2 after grouping all the binding sites belonging to the same class of transcription factor, only four binding sites are common to all the genomic fragments active in the sensory vesicle (white background). Furthermore, GR, HSF and LEF-1 sites are also present in the other mouse sequences that are not active in *Ciona *sensory vesicle (grey background). The only binding site common to all the sequences expressed in the sensory vesicle, but not detected in the other regulatory elements is *Pax6*. *Pax6 *is a transcription factor expressed in the sensory vesicle of *Ciona *embryos but it does not seem to be involved in *Hox *gene regulation. It is possible that the position of the Pax6 binding sites in regions that exert primary roles in controlling the expression profile of these *Hox *genes has led to their conservation in *Ciona *and vertebrates.

**Table 2:** Binding sites common to all the regulatory sequences active in sensory vesicle

Binding site	1UP1.4	4UP1.3	2D0.8	1CiHox3	mb1	mb2	ma2	ma3	mb1 RARE
**GR**	**2**	**3**	**4**	**3**	**1**	4	2	0	1

**HSF**	**2**	**5**	**5**	**2**	**3**	2	2	1	0

**LEF1**	**2**	**1**	**1**	**1**	**2**	3	0	1	1

**Pax6**	**8**	**4**	**3**	**5**	**1**	0	0	0	0

*Ciona *and mouse regulatory sequences active in the sensory vesicle (bold character) compared with the dataset not expressed in the sensory vesicle (light character).

When analyzing the binding sites common to amphioxus *2B *and *Ciona 2D0.5 *elements, we identified several Ets sites in the *2B *sequence. However, these motifs were not detected in the *2D0.5 *sequence when minimizing the error rates for both the false positive and the false negative matches. Therefore we reanalysed the *2D0.5 *sequence using different parameter settings [43]. In this case, the Ets binding sites were now detected in the *2D0.5 *fragment but, the Ets identified on the *2B *sequence do not correspond to the region characterized as functionally active [42]. We then compared these two sequences considering non redundant patterns as parameter settings but, again, the patterns in the *2B *sequence do not correspond to the functional Ets sites.

These results suggest that non canonical Ets sites are present in the *2B *sequence and that, despite the perfect coincidence of expression of the *Ciona *and amphioxus *Hox2 *genomic fragments, the low levels of sequence conservation do not permit the unambiguous identification of the corresponding functional sequence(s) in the *Ciona 2D0.5 *element.

The expression of *Ciona CiHox1 *(*1UP1.4*) and *CiHox3 *(*1CiHox3*) regulatory sequences in rhombomere 4 of transgenic vertebrates reproduced the profile of Hox/Pbx regulatory complexes common to mouse *mb1, mb2 *and *ma2 *enhancers. We then, searched these *Ciona *sequences for possible Hox/Pbx/Meis binding sites and compared them with that of mouse r4 specific elements present in the *mb1, mb2 *and *ma2 *elements. We then, also analyzed and compared the regulatory sequences not specifically active in rhombomere 4 (see Additional File 2: Table S2). This analysis identified several conserved and significant binding sites. The majority of them are not specific for the sequences expressed in rhombomere 4, but are also present in the other sequences analyzed. Interestingly, as shown in Table 3 among all the Pbx and Meis binding sites identified, the Pbx and Pbx-1b (bold) could be of particular relevance. They are, in fact, responsible for mouse *Hoxb1 *specific expression in rhombomere 4 [35,37] and are present in both the *1UP1.4 *and *1CiHox3 *sequences but are absent in the *1intro1.7 *sequence that is not active in r4.

**Table 3:** Pbx and Meis binding sites in *Ciona *and mouse regulatory sequences

Binding site	1UP1.4	4UP1.3	2D0.8	1CiHox3	1intro1.7	mb1	mb2	ma2	ma3	mb1RARE
**pbx-1b**	**3**	**1**	**2**	**3**	**0**	**2**	**2**	**1**	**0**	**0**

Pbx-1	7	3	2	4	2	1	3	1	4	0

**Pbx**	**1**	**1**	**0**	**1**	**0**	**4**	**1**	**2**	**0**	**0**

MEIS1A	2	2	1	6	1	0	4	1	3	0

MEIS1	0	1	0	2	0	0	4	1	0	0

MEIS1B	0	0	0	0	0	0	1	0	0	0

With light characters are indicated the regulatory sequences that do not contain or do not show functionally active Pbx/Meis elements.

## Discussion

Genome sequencing from a variety of vertebrate and invertebrate organisms revealed that the evolution of more complex structures is mostly due not only to the increase in gene numbers but also to the acquisition of novel regulatory circuits and as consequence of novel functions by preexisting genes [20]. Under this view, the conservation or modification of cis-regulatory elements controlling genes that exert primary roles during embryonic development can help to explain how vertebrate innovations have been acquired. Of particular interest is to study the genes involved in the formation of anterior neural structures where the major differences can be observed between vertebrates and other chordates. Vertebrates are characterized by both morphologic and genetic key characters of the cephalic structures and try to understand the mechanisms that led to the evolution of these neural structures in the chordate lineage represents an intriguing challenge.

We have compared neural specific regulatory elements of the anterior *Hox *genes from three different chordate species, the cephalochordate amphioxus, the urochordate *Ciona intestinalis *and the vertebrate mouse to begin to understand the mechanisms that led to the evolution of neural structures in the chordate lineage. In this study we have identified and characterized *cis*-regulatory regions implicated in controlling the neural expression of *Ciona Hox *genes from paralogous groups 1-4. Together with our previous work on *CiHox3 *[15], we find evidence for 5 regulatory regions that appear to recapitulate most of the endogenous expression patterns for *CiHox1, CiHox3 *and *CiHox4 *(Figures 1, 2) and are specifically active in the nervous system. We made direct comparisons of *cis*-regulatory regions from the *Ciona*, amphioxus and mouse *Hox *genes in paralogous groups 1-4 to explore the degree to which their *cis*-regulatory information has been conserved during evolution. We experimentally tested the ability of various anterior *Hox *regulatory elements from amphioxus and mouse to function in *Ciona *embryos and *Ciona *fragments to work in vertebrate embryos. This strategy permitted the identification of conserved sequences and apparently non conserved sequences that were able to elicit the same functions in different species. In addition, this functional comparison permitted the identification of regulatory regions that would have been impossible to identify only on the bases of sequence comparison. Our findings lead to several general observations that have interesting implications for understanding mechanisms that underlie the control of *Hox *expression in generating regional characteristics in the anterior nervous system. These issues will be discussed below.

*Cis*-regulatory modules that receive input from transcription factors, such as Krox20 and Kreisler, play key roles in mediating segment-specific activation of *Hox *genes in the vertebrate hindbrain. These elements have not been identified by in silico analysis in *Ciona *and amphioxus *Hox1*-3 regulatory regions. Furthermore, the mouse Krox20 and Kreisler enhancers present in *ma2, mb2 *and *ma3 *fragments were not functionally active in *Ciona *embryos. The same situation occurred with the mouse *Hoxa3 *and *Hoxb3 *enhancers tested by Locascio et al. [15], where the Kreisler sites have not been recognized by the *Ciona *regulatory machinery. These results, together with the observation that amphioxus anterior *Hox *genomic fragments are not able to reproduce in mouse and chicken embryos any Kreisler or Krox specific expression profile [23], indicate that this mode of activating early *Hox *expression in the anterior CNS is not present in *Ciona *or amphioxus and is specific for the vertebrate lineage. It appears that *Ciona*, amphioxus and vertebrates utilize different sets of factors from each other to initiate or establish their early domains of *Hox *expression, which may reflect differences in their respective embryogenic processes. However, it is possible that there are some overlaps in the signalling pathways that directly initiate *Hox *expression or different upstream factors that in turn activate *Hox *expression in *Ciona*, amphioxus and vertebrates. For example, retinoid signalling directly activates vertebrate group 1 and group 4 *Hox *genes through RAREs positioned near the genes [40,44-46], and *AmphiHox1 *contains RAREs that activate its expression [22,23]. *AmphiHox1*, 2, 3 and 4 collinear expression in the CNS has been demonstrated to be controlled by RA-signalling [5]. Furthermore, amphioxus *Hox *regulatory elements studied in mouse and chicken embryos evidenced the existence of conserved retinoic acid dependent neural elements [22,23]. In *Ciona*, only an epidermal RARE element has been identified in the *CiHox1 *gene [29] and we have not found RAREs in the nervous specific regulatory regions of *Ciona CiHox1-4 *genes. In addition, mouse RARE elements contained in the *mb1RARE *and *md4 *fragments do not direct reporter expression in *Ciona *(data not shown). This is consistent with reports that *Ciona *embryos may have a very reduced ability to respond to retinoic acid [22,27,28]. In vertebrates, retinoid, FGF and Wnt signalling can all serve as posteriorizing influences to modulate *Hox *expression, and the relative degree to which any one or combination of these pathways contribute to *Hox *regulation can vary between species. Therefore, it will be important to assess the degree to which inputs from these three pathways may be implicated in regulating initial *Hox *expression in *Ciona*, amphioxus and vertebrates.

Despite the differences in activation of *Hox *expression, our analyses indicate that auto- and cross-regulatory inputs from *Hox *genes themselves is a conserved mechanism for maintaining patterns of *Hox *expression only in *Ciona *but not in amphioxus embryos. Regulatory regions from *CiHox1 *and *CiHox3*, which recapitulate endogenous *Ciona *expression, serve as *Hox *response elements. The *Ciona CiHox1 (1UP1.4) *and *CiHox3 (1CiHox3) *regulatory sequences when tested in mouse and chicken embryos generate reporter expression in rhombomere 4 in a manner similar to the mouse *mb1, mb2 *and *ma2 *enhancers (Table 1). These three mouse enhancers have been shown to serve as *Hox *response elements dependent upon the binding of Hox/Pbx and Meis complexes. Furthermore, when the mouse *mb1 *enhancer is tested in *Ciona *it directs reporter expression in a pattern similar to endogenous *CiHox1 *expression (Figures 6A, B). We experimentally demonstrated that *Ciona 1UP1.4 *expression is under the control of Hoxb1/Pbx regulatory complexes (Figures 7A, B). Furthermore, in silico comparison of the *Ciona *sequences reveals multiple Hox/Pbx/Meis binding sites, similar to those found in the r4 specific regulatory elements from mouse (Table 3). As control, we also analysed the other mouse enhancers tested in this study that do not contain functional Hox/Pbx binding sites and the *Ciona *elements that are not expressed in rhombomere 4 (grey background). Together, these data underscore the important and conserved role that auto and cross-regulation plays in regulating *Hox *expression in the *Ciona *nervous system. Since Hox proteins can serve to both activate and repress activity, such *Hox *response elements might be integrating inputs from multiple *Hox *genes to maintain their restricted patterns following initial activation by different upstream factors.

Most of the amphioxus *Hox1-3 *regulatory sequences tested in *Ciona *embryos did not work at all. The *2B *fragment, the only one active in *Ciona *embryos, directed expression in the anterior CNS but not in a manner reminiscent of a segment specific profile that is typical of Hox/Pbx regulatory complexes. These amphioxus elements have also been tested in vertebrates, but again, none of them was able to direct any r4 or segment specific expression in chicken or mouse embryos [23]. Despite the lacking of specific experiments with amphioxus *Hox*1-3 elements and Hox/Pbx complexes, considering the evolutionary position of cephalochordates at the base of chordate origin, these results seem to indicate that this auto and cross-regulatory mechanism is not present in cephalochordates and thus appeared later in evolution after their divergence but before the appearance of urochordates.

Among all the *Hox *genomic fragments tested by electroporation it is evident that the *Ciona 1UP1.4, 2D0.8, 4UP1.3, 1CiHox3 *and mouse *mb1 *genomic fragments contain regulatory elements specifically active in the CNS of *Ciona *embryos at level of the sensory vesicle or of its precursors (Table 1). This expression is not specific to any *Ciona Hox *gene and illustrates the presence of multiple elements interspersed among all the anterior *Ciona Hox *genes and conserved up to vertebrates. The in silico analysis of these genomic fragments showed that a particular Pax6 binding site is present in all these sequences. All the other *Ciona *and mouse fragments that fail to direct reporter expression in the *Ciona *sensory vesicle lack this recognition sequence, suggesting that it is important for regulatory activity.

*Pax6 *in *Ciona *embryos, is expressed in the sensory vesicle and in its precursors at tailbud stage [47] and is therefore a good candidate to explain the common expression profile of these *Ciona *and mouse elements. Considering that the sensory vesicle does not correspond to any *Ciona Hox *specific territory of expression, these elements may become active only when extracted from their natural context, suggesting that repressor elements in the genes normally silence these elements.

## Conclusions

We have compared the activity of four *Ciona cis*-elements located near the *CiHox1, CiHox2 *and *CiHox4 *genes and of a *CiHox3 *element previously identified [15] with the amphioxus *Hox1-3 *elements and a series of mouse neural specific enhancers from *Hox *paralogous groups 1-4 which direct segmental expression in the developing hindbrain. The regulatory potential of all fragments were tested in transgenic *Ciona *embryos and in addition the *Ciona CiHox1, CiHox2 *and *CiHox4 *elements have been assayed in chicken embryos for their ability to be recognised by the vertebrate transcriptional machinery.

We found that segment-specific neural enhancers from mouse *Hox2 *and *Hox3 *genes dependent upon Krox20 and kreisler for activity are not functional in *Ciona*. Using the regulatory regions functionally identified in *Ciona *and chicken embryos, we used sequence analyses to compare the enhancer fragments located in similar positions which generated related expression profiles. This approach revealed that some enhancers serve as *Hox *response elements through the action of Hox/Pbx binding motifs. Hence, these enhancers contain *cis*-elements able to reproduce or maintain the segmental expression patterns typical of *Hox *genes through cross- and auto-regulatory influences of the Hox proteins themselves. This component of *Hox *regulation has been conserved during chordate evolution and the functional activity reveals that the *cis*-elements are recognized by the regulatory components and mechanisms of different and extant species.

In summary, our study together with previous studies on chordate retinoic acid dependent regulatory regions [22-24,29] suggests that, during *Hox *cluster evolution, retinoic acid responsive elements were already present in the basal chordate ancestor. They have been maintained from amphioxus to vertebrate [22,24] but extremely reduced and restricted to epidermal tissues in urochordates [22,29]. Auto- and cross-regulatory elements, that direct segment specific expression in the CNS under the control of Hox/Pbx and Prep/Meis complexes seem to be not present in amphioxus and may have appeared later with urochordates. These have then been conserved in vertebrates, although accompanied with extensive rearrangements and modifications. Finally, the Krox20 and Kerisler/Mafb responsive elements responsible for early *Hox *gene activation and for highly specific and coordinated expression in vertebrate hindbrain seem to have evolved along the chordate lineage after urochordates divergence.

## Methods

### Ascidians and embryos

*Ciona intestinalis *adults were collected in the Bay of Naples and cultured by the Marine Resources for Research Service of the Stazione Zoologica. Embryos were raised in filtered sea water at 16°-18°C and samples at appropriate stages of development were fixed for whole mount in situ hybridization in 4% paraformaldehyde and dehydrated in ethanol series.

Ascidians are non-vertebrate chordate animals and according to the European committement their manipulation does not need any ethic committee approval.

All experimental work involving vertebrate animals was performed according to a project and procedures # 2010-0062 approved by the Stowers Institute Animal Care and Usa Committee (IACUC).

### Preparation of *Ciona *constructs

The basic electroporation vectors were pBlueScript II KS containing the *lacZ *and SV40 polyadenylation sequences downstream of the human *β-globin *or the *CiHox3 *0.2 basal promoters [15]. The *CiHox1 *genomic fragments 1UP2.4, 1intro3.6 and 1D1.7 were amplified by PCR using as template the cosmid clone MPMGc119H2170 isolated from a cosmid library prepared by the Reference Library Database [48]. The fragments 1UP2.1 and 1UP3.0 were obtained by digestion from the same cosmid clone by using the *Xba*I-*Sma*I and *Eco*RI-*Hind*III restriction enzymes respectively.

The genomic fragments 2UP4.2 and 2D3.1 were obtained by digestion of the cosmid clones MPMGc119C0437 and MPMGc119L0224. The fragments 2UP2.2 and 2D1.4 were amplified by PCR from the same cosmid clones.

Among the genomic fragments of *CiHox4 *gene, only the 4UP 3.4 and the 4D2.7 were obtained by digestion of the cosmid clones MPMGc119D1338 and MPMGc119B114 respectively. All the other fragments, 4UP2.1, 4D1.2, 4D3.4 and 4D3.7, were amplified by PCR using the same cosmid clones as template.

All the deleted fragments were obtained by digestion or PCR amplification of the corresponding larger fragments.

All the constructs have been controlled by sequence analysis prior to use. Further information on the constructs and the oligonucleotide sequences used for PCR amplifications are available upon request.

### Preparation of mouse and amphioxus constructs

All the mouse genomic fragments corresponding to: the *Eco*RI-*Hae*III insert of construct #15 [35] and the *Eco*RV-*Hind*III insert of construct #9 [39] of the *Hoxb1 *gene; the *Bgl*II insert of construct #1 of the *Hoxa2 *gene [38]; the *Bam*HI-*Eco*RI insert of 2.1 kb of the *Hoxb2 *gene [37]; the *Eco*RV-*Ava*II insert of construct #1 of the *Hoxa3 *gene; the insert of construct #16 of the *Hoxd4 *gene [40], were cloned in the basic electroporation vectors described in "preparation of *Ciona *constructs".

The amphioxus genomic fragments corresponding to the 1A, 1B, 2A, 2B, 2C, 3A and 3B constructs [23] were prepared as described for the mouse fragments. As for the *Ciona *constructs also all the mouse and amphioxus constructs have been controlled by sequence analysis prior to use.

### *Ciona *embryo electroporation

All the constructs purified on CsCl gradient were electroporated into dechorionated and fertilized *Ciona *eggs and assayed for LacZ expression as described [15]. Each construct has been assayed at least five times in separated experiments and hundreds of *Ciona *embryos have been analysed in each experiment. LacZ staining has been considered specific when reproducing always the same pattern in at least 80% of positive embryos while has been considered unspecific when the expression was so variable that it was not possible to define its extension in a given territory. LacZ expression that did not reproduce endogenous gene expression has been termed ectopic. In all the diagrams the double plus indicates a signal that appears in less than 24 hours of β-galactosidase staining, while the single plus indicates a signal visible after more than 24 hours.

The *Ciona CiHox3 *0.2 basal promoter sequence is not able alone to activate any LacZ expression. The human *β-globin *basal promoter activates ectopic LacZ expression only in the mesenchyme.

Gelatin embedded sections were performed as described [49]. Embryos and sections were photographed by using a Zeiss AxioImager M1 microscope.

### Chicken embryo electroporation

*In ovo *electroporation of chicken embryos was performed as previously described [50] and [51]. Plasmid DNA, prepared from BGZ40 [34] containing *Ciona *fragments, was co-injected with Fast Green and CMV-GFP control plasmid (2.5 μg/ul) into the neural tube of Hamburger-Hamilton (HH) stage 4 or 8-10 chicken embryos. Embryos were electroporated with DNA, allowed to develop *in ovo *for an additional 18-20 hours, and viewed under a fluorescent dissecting microscope to screen for GFP expression, indicating successful electroporation and expression. Embryos showing GFP expression were stained for β-galactosidase activity. Co-electroporations were performed as described above with the *Hoxb1 *expression vector added at a final concentration of 2.5 μg/μl. The mouse mb1 region was used as a positive control for these experiments [52].

### In situ hybridization

The *CiHox1, CiHox4 *and *Stac *cDNA clones were from the *Ciona *gene collection of the Ghost Database, ID: citb014p24, ciad029a11 and ciad017p01 respectively [53]. Whole mount in situ hybridizations were performed as described [54].

### In silico analysis

We analysed the potential binding sites for transcription factors using the Match tool [55]. Match uses a library of mononucleotide weight matrices from the database TRANSFAC 11.4 professional [43]. We used the cut-off to minimize the error rates of both the false positive and false negative matches. To do so, the software computes the number of matches found in promoter sequences for each matrix using a cut-off allowing 10% of false negative matches (minFN10). This number is defined as 100% of false positives. The sum of corresponding percentages for false positives and false negatives is then computed for every cut-off ranging from minFN10 to minFP. We refer to the cut-off that gives the minimum sum as minSum cut-off.

The analyses were performed using an "in house" software developed by the CAB group [56] of the University of Naples "Federico II". The software permits the reconciliation of the results from the Match tools and the detection of common binding sites among sequences.

## List of abbreviations

CNS: central nervous system; e: epidermis; mp: mesenchymal pockets; nc: nerve cord; ns: nervous system; p: palps; ph: pharynx; sv: sensory vesicle; tlc: trunk lateral cells; tsn: tail sensory neurons; vg: visceral ganglion.

## Authors' contributions

AN, AM and ED prepared all the *Ciona *constructs and AN also performed the electroporation experiments in *Ciona *embryos. CS performed all the experiments in chicken embryos and MLC was responsible for the in silico analyses. MB and LF discussed the results, RK directed the studies in chicken embryos and supervised manuscript organization and revision, AL coordinated the project and wrote the manuscript. All authors read and approved the final manuscript.

## Supplementary Material

Additional file 1 **Table S1. Binding site consensus common to all the regulatory sequences active in sensory vesicle**. List of the specific binding site consensus together with the transcription factor classes they belong to and their occurrence in the *Ciona *and mouse regulatory sequences. Click here for file

Additional file 2 **Table S2. Pbx and Meis consensus binding sites in *Ciona *and mouse regulatory sequences**. Distribution of binding sites consensus from the Pbx and Meis classes in the *Ciona *and mouse regulatory sequences. Click here for file
