# Supplementary material for: Evolution of anterior Hox regulatory elements among chordates
Source: BMC Evol Biol. 2011 Nov 15;11:330. doi: 10.1186/1471-2148-11-330 (PMC3227721; doi:10.1186/1471-2148-11-330)
Supplement: Additional file 1 — Table S1. Binding site consensus common to all the regulatory sequences active in sensory vesicle. List of the specific binding site consensus together with the transcription factor classes they belong to and their occurrence in the Ciona and mouse regulatory sequences. [file 1471-2148-11-330-S1.DOC]

**Additional File 1:**

**Table S1: Binding site consensus common to all the regulatory sequences** active in sensory vesicle

| TF class | consensus | 1UP1.4 | 4UP1.3 | | 2D0.8 | | 1CiHox3 | | mb1 | | mb2 | | ma2 | | ma3 | | mb1RARE | | **ROW SUM** | |
| --- | --- | --- | --- | --- | --- | --- | --- | --- | --- | --- | --- | --- | --- | --- | --- | --- | --- | --- | --- | --- |
| GR | aaaGAACAgaacgctatgc | 0 | 0 | | 1 | | 0 | | 0 | | 0 | | 0 | | 0 | | 0 | | **1** | |
| GR | tcgtaccccacTGTACtat | 0 | 0 | | 0 | | 1 | | 0 | | 0 | | 0 | | 0 | | 0 | | **1** | |
| GR | tgaGTACAgatgaccccaa | 0 | 0 | | 0 | | 0 | | 0 | | 1 | | 0 | | 0 | | 0 | | **1** | |
| GR | aaaattaGAACAacgtggttctattta | 0 | 1 | | 0 | | 0 | | 0 | | 0 | | 0 | | 0 | | 0 | | **1** | |
| GR | ctgcctctttcTGTCCttg | 0 | 0 | | 0 | | 0 | | 0 | | 1 | | 0 | | 0 | | 0 | | **1** | |
| GR | tagtaaaGAACAtttacttagcatgga | 0 | 0 | | 0 | | 1 | | 0 | | 0 | | 0 | | 0 | | 0 | | **1** | |
| GR | aagctgagatgTGTTCtta | 0 | 0 | | 0 | | 0 | | 0 | | 0 | | 1 | | 0 | | 0 | | **1** | |
| GR | cattaaaGAACAgaacgctatgcgaag | 0 | 0 | | 1 | | 0 | | 0 | | 0 | | 0 | | 0 | | 0 | | **1** | |
| GR | aggaacctgttTGTTCttc | 1 | 0 | | 0 | | 0 | | 0 | | 0 | | 0 | | 0 | | 0 | | **1** | |
| GR | tgtcctaGAACAccatcccaagacaaa | 0 | 0 | | 0 | | 0 | | 1 | | 0 | | 0 | | 0 | | 0 | | **1** | |
| GR | acaGGACAgaaagcgactg | 0 | 1 | | 0 | | 0 | | 0 | | 0 | | 0 | | 0 | | 0 | | **1** | |
| GR | atataggaacctgttTGTTCttcatcg | 1 | 0 | | 0 | | 0 | | 0 | | 0 | | 0 | | 0 | | 0 | | **1** | |
| GR | ttaGAACAacgtggttcta | 0 | 1 | | 0 | | 0 | | 0 | | 0 | | 0 | | 0 | | 0 | | **1** | |
| GR | ttaGAACAcctcgcattaa | 0 | 0 | | 1 | | 0 | | 0 | | 0 | | 0 | | 0 | | 0 | | **1** | |
| GR | gaaGGACAgattaatagat | 0 | 0 | | 0 | | 1 | | 0 | | 0 | | 0 | | 0 | | 0 | | **1** | |
| GR | cttgaagctgagatgTGTTCttaaggg | 0 | 0 | | 0 | | 0 | | 0 | | 0 | | 1 | | 0 | | 0 | | **1** | |
| GR | tggacagtggGTCCTg | 0 | 0 | | 0 | | 0 | | 0 | | 0 | | 0 | | 0 | | 1 | | **1** | |
| GR | acaGGACAgtctgggctct | 0 | 0 | | 0 | | 0 | | 0 | | 1 | | 0 | | 0 | | 0 | | **1** | |
| GR | gtgattaGAACAcctcgcattaatcaa | 0 | 0 | | 1 | | 0 | | 0 | | 0 | | 0 | | 0 | | 0 | | **1** | |
| GR | cAGGACagtctgggct | 0 | 0 | | 0 | | 0 | | 0 | | 1 | | 0 | | 0 | | 0 | | **1** | |
|  |  |  |  | |  | |  | |  | |  | |  | |  | |  | |  | |
| HSF | AGAAC | 0 | 2 | | 5 | | 2 | | 2 | | 0 | | 0 | | 0 | | 0 | | **22** | |
| HSF | GTTCT | 2 | 3 | | 0 | | 0 | | 1 | | 2 | | 2 | | 1 | | 0 | | **16** | |
| HSF2 | CGAATcttcg | 0 | 0 | | 0 | | 0 | | 0 | | 0 | | 0 | | 1 | | 0 | | **1** | |
| HSF2 | cgaatCTTCG | 0 | 0 | | 0 | | 0 | | 0 | | 0 | | 0 | | 1 | | 0 | | **1** | |
| LEF1 | tCAAAG | 1 | 1 | | 1 | | 0 | | 0 | | 0 | | 0 | | 1 | | 0 | | **7** | |
| LEF1 | CTTTGa | 1 | 0 | | 0 | | 1 | | 2 | | 3 | | 0 | | 0 | | 1 | | **10** | |
| Pax-6 | tttaacttcagCGCGAaaccg | 0 | 1 | | 0 | | 0 | | 0 | | 0 | | 0 | | 0 | | 0 | | **1** | |
| Pax-6 | aaattTTACGgttgaatattt | 0 | 0 | | 1 | | 0 | | 0 | | 0 | | 0 | | 0 | | 0 | | **1** | |
| Pax-6 | cccacTCACGaatcaactttc | 1 | 0 | | 0 | | 0 | | 0 | | 0 | | 0 | | 0 | | 0 | | **1** | |
| Pax-6 | tcgctTGAGGtttcaattgtg | 0 | 1 | | 0 | | 0 | | 0 | | 0 | | 0 | | 0 | | 0 | | **1** | |
| Pax-6 | tctatgggatgCGTGAgcaat | 1 | 0 | | 0 | | 0 | | 0 | | 0 | | 0 | | 0 | | 0 | | **1** | |
| Pax-6 | attttTCTTGgataattttta | 0 | 0 | | 0 | | 1 | | 0 | | 0 | | 0 | | 0 | | 0 | | **1** | |
| Pax-6 | tagtatttatgAGTTAataga | 1 | 0 | | 0 | | 0 | | 0 | | 0 | | 0 | | 0 | | 0 | | **1** | |
| Pax-6 | cggctTTATGcctaaacttcc | 1 | 0 | | 0 | | 0 | | 0 | | 0 | | 0 | | 0 | | 0 | | **1** | |
| Pax-6 | gttttTGACGcttccatgtcg | 0 | 0 | | 0 | | 0 | | 1 | | 0 | | 0 | | 0 | | 0 | | **1** | |
| Pax-6 | acggctttatgCCTAAacttc | 1 | 0 | | 0 | | 0 | | 0 | | 0 | | 0 | | 0 | | 0 | | **1** | |
| Pax-6 | tttttTCAAGcgtttttttaa | 0 | 0 | | 0 | | 1 | | 0 | | 0 | | 0 | | 0 | | 0 | | **1** | |
| Pax-6 | ttactTAATGgattattctat | 0 | 0 | | 0 | | 1 | | 0 | | 0 | | 0 | | 0 | | 0 | | **1** | |
| Pax-6 | accctttcaaaCGTCAcaaag | 0 | 0 | | 0 | | 1 | | 0 | | 0 | | 0 | | 0 | | 0 | | **1** | |
| Pax-6 | caattTGACGctttggtattt | 0 | 0 | | 1 | | 0 | | 0 | | 0 | | 0 | | 0 | | 0 | | **1** | |
| Pax-6 | agttagtgagaCATAAatggt | 0 | 0 | | 1 | | 0 | | 0 | | 0 | | 0 | | 0 | | 0 | | **1** | |
| Pax-6 | atatattcactCGGTAatatt | 1 | 0 | | 0 | | 0 | | 0 | | 0 | | 0 | | 0 | | 0 | | **1** | |
| Pax-6 | aaattTTATGattgatttatg | 1 | 0 | | 0 | | 0 | | 0 | | 0 | | 0 | | 0 | | 0 | | **1** | |
| Pax-6 | atcctTGTTGcattagtttag | 1 | 0 | | 0 | | 0 | | 0 | | 0 | | 0 | | 0 | | 0 | | **1** | |
| Pax-6 | gtaaaataaacCTGGAaaacg | 0 | 1 | | 0 | | 0 | | 0 | | 0 | | 0 | | 0 | | 0 | | **1** | |
| Pax-6 | caaaaTGGCGgttgagttgaa | 0 | 1 | | 0 | | 0 | | 0 | | 0 | | 0 | | 0 | | 0 | | **1** | |
| Pax-6 | gaattTCAAGtttaattttga | 0 | 0 | | 0 | | 1 | | 0 | | 0 | | 0 | | 0 | | 0 | | **1** | |
| TOTAL |  |  |  | |  | |  | |  | |  | |  | |  | |  | |  | |
| GR |  | 2 | 3 | | 4 | | 3 | | 1 | | 4 | | 2 | | 0 | | 1 | | 20 | |
| HSF |  | 2 | 5 | | 5 | | 2 | | 3 | | 2 | | 2 | | 1 | | 0 | | 27 | |
| LEF1 |  | 2 | 1 | | 1 | | 1 | | 2 | | 3 | | 0 | | 1 | | 1 | | 12 | |
| PAX6 |  | 8 | 4 | | 3 | | 5 | | 1 | | 0 | | 0 | | 0 | | 0 | | 21 | |
|  |  |  | |  | |  | |  | |  | |  | |  | |  | |  | |  |

List of the specific binding site consensus together with the transcription factor classes they belong to and their occurrence per analyzed sequence.

Total number of matches per transcription factor class are also reported at the end of the table. Upper case in the consensus indicates the most conserved residues in the weight matrix defining the binding site.
