# Supplementary material for: Evolution of anterior Hox regulatory elements among chordates
Source: BMC Evol Biol. 2011 Nov 15;11:330. doi: 10.1186/1471-2148-11-330 (PMC3227721; doi:10.1186/1471-2148-11-330)
Supplement: Additional file 2 — Table S2. Pbx and Meis consensus binding sites in Ciona and mouse regulatory sequences. Distribution of binding sites consensus from the Pbx and Meis classes in the Ciona and mouse regulatory sequences. [file 1471-2148-11-330-S2.DOC]

**Additional File 2:**

**Table S2: Pbx and Meis consensus binding sites in *Ciona* and mouse regulatory sequences**

| Sequence ID | TF class | Consensus | strand | position | core similarity | matrix similarity | |
| --- | --- | --- | --- | --- | --- | --- | --- |
|  | Pbx-1b | atattGATTGagcat | (-) | 78 | 1 | 0,902 |  |
|  | Pbx-1 | attgaTTGAGca | (-) | 80 | 0,9 | 0,914 |  |
| 2D0.8 | MEIS1A:HOXA9 | TGGCAggttatgag | (+) | 148 | **0,821** | **0,755** |  |
|  | Pbx-1b | cgcatTAATCaactt | (+) | 420 | 0,789 | 0,859 |  |
|  | Pbx-1 | attAATCAa | (+) | 423 | **1** | **0,981** |  |
|  | Pbx | agcccttCCATC | (-) | 188 | 0,942 | 0,914 |  |
|  | MEIS1A:HOXA9 | gccagaagcTGTCA | (-) | 363 | 1 | 0,839 |  |
| ma2 | MEIS1 | aagcTGTCAggg | (-) | 368 | **1** | **0,997** |  |
|  | Pbx-1b | agcaaGATTGatcac | (-) | 389 | 1 | 0,842 |  |
|  | Pbx-1 | caagaTTGATca | (-) | 391 | **1** | **0,938** |  |
|  | Pbx | GATTGatcacac | (+) | 394 | 1 | 0,918 |  |
|  | Pbx-1 | aaaAATCAa | (+) | 25 | 1 | 0,956 |  |
|  | Pbx-1b | aaaatCAATAaaatc | (+) | 26 | 0,789 | 0,856 |  |
|  | Pbx-1 | atcAATAAa | (+) | 29 | 0,948 | 0,961 |  |
|  | Pbx-1b | tttatGATTGattta | (-) | 75 | 1 | 0,927 |  |
|  | Pbx-1 | tatgaTTGATtt | (-) | 77 | **1** | **0,997** |  |
|  | Pbx-1 | aTGATTgat | (-) | 78 | 1 | 0,962 |  |
| 1UP1.4 | MEIS1A:HOXA9 | TGATTgatttatga | (+) | 79 | 0,652 | 0,817 |  |
|  | Pbx-1b | tgattGATTTatgat | (-) | 79 | 0,789 | 0,858 |  |
|  | Pbx | GATTGatttatg | (+) | 80 | 1 | 0,943 |  |
|  | Pbx-1 | tTGATTtat | (-) | 82 | 1 | 0,97 |  |
|  | MEIS1A:HOXA9 | cttttaaccTGTTA | (-) | 129 | **0,821** | **0,796** |  |
|  | Pbx-1 | acgAATCAa | (+) | 356 | 1 | 0,963 |  |
|  | Pbx-1 | tgtaaTTGATaa | (-) | 415 | 1 | 0,92 |  |
|  | MEIS1 | aaaTGACAgcga | (+) | 10 | **1** | **0,997** |  |
|  | MEIS1A:HOXA9 | TAACAgttttgcca | (+) | 42 | 0,821 | 0,82 |  |
| 4UP1.3 | Pbx-1 | aatAATCAa | (+) | 57 | 1 | 0,968 |  |
|  | Pbx-1b | gatttGATTAatttg | (-) | 151 | 0,789 | 0,859 |  |
|  | Pbx-1 | tTGATTaat | (-) | 154 | **1** | **0,981** |  |
|  | Pbx | GATTGaggtgtc | (+) | 417 | 1 | 0,912 |  |
|  | Pbx-1 | attAATCAt | (+) | 575 | 1 | 0,943 |  |
|  | MEIS1A:HOXA9 | TAACAgataacgga | (+) | 743 | 0,821 | 0,759 |  |
|  | MEIS1A:HOXA9 | TGGCAggtgtattt | (+) | 113 | 0,821 | 0,744 |  |
|  | MEIS1A:HOXA9 | GGACAgattaatag | (+) | 135 | 0,738 | 0,764 |  |
|  | Pbx-1b | attttGATGGatata | (-) | 246 | 0,855 | 0,897 |  |
|  | Pbx-1 | tttgaTGGATat | (-) | 248 | 0,893 | 0,911 |  |
|  | Pbx-1 | tTTATTgct | (-) | 308 | 0,948 | 0,942 |  |
|  | MEIS1 | ttgTGACAgtga | (+) | 665 | **1** | **0,997** |  |
|  | MEIS1A:HOXA9 | TGGCAgttgacaaa | (+) | 756 | 0,821 | 0,742 |  |
|  | Pbx-1b | gctttGATGGatgga | (-) | 1446 | 0,855 | 0,895 |  |
| 1CiHox3 | Pbx-1 | tttgaTGGATgg | (-) | 1448 | 0,893 | 0,913 |  |
|  | Pbx | GATGGatggagc | (+) | 1451 | 0,942 | 0,953 |  |
|  | MEIS1A:HOXA9 | ggatggagcTGTCA | (-) | 1454 | 1 | 0,788 |  |
|  | MEIS1 | gagcTGTCAata | (-) | 1459 | **1** | **0,998** |  |
|  | MEIS1A:HOXA9 | TTACAggttaacct | (+) | 1479 | 0,805 | 0,782 |  |
|  | Pbx-1b | ccgatCAAGCaaaat | (+) | 1912 | 0,789 | 0,857 |  |
|  | Pbx-1 | cgATCAAgcaaa | (+) | 1913 | **1** | **0,969** |  |
|  | MEIS1A:HOXA9 | TGAGCgtttacgta | (+) | 2128 | 0,696 | 0,733 |  |
|  | Pbx | GATGGatgggct | (+) | 435 | 0,942 | 0,95 |  |
| mb1 | Pbx-1b | agagtGATTGaagtg | (-) | 448 | 1 | 0,847 |  |
|  | Pbx | GAGTGattgaag | (+) | 449 | 0,887 | 0,908 |  |
|  | Pbx | GATTGaagtgtc | (+) | 453 | **1** | **0,921** |  |
|  | Pbx-1b | ggggtGATGGatggg | (-) | 485 | 0,855 | 0,831 |  |
|  | Pbx-1 | ggtgaTGGATgg | (-) | 487 | 0,893 | 0,912 |  |
|  | Pbx | GATGGatgggcg | (+) | 490 | 0,942 | 0,959 |  |
|  | MEIS1A:HOXA9 | cataaaatcTGTCA | (-) | 48 | 1 | 0,846 |  |
|  | MEIS1 | aatcTGTCAtca | (-) | 53 | 1 | 0,996 |  |
|  | MEIS1 | cacTGACAgaaa | (+) | 499 | **1** | **0,998** |  |
|  | MEIS1A:HOXA9 | ggagggagcTGTCA | (-) | 806 | 1 | 0,745 |  |
|  | MEIS1 | gagcTGTCAggg | (-) | 811 | **1** | **0,998** |  |
|  | Pbx-1b | gctaaGATTGatcgc | (-) | 824 | 1 | 0,84 |  |
|  | Pbx-1 | taagaTTGATcg | (-) | 826 | 1 | 0,94 |  |
|  | Pbx | GATTGatcgcct | (+) | 829 | **1** | **0,956** |  |
| mb2 | MEIS1A:HOXA9 | TGAAAgttaagtgg | (+) | 1262 | 0,805 | 0,735 |  |
|  | MEIS1A:HOXA9 | gaatacatcTGTCA | (-) | 1327 | 1 | 0,822 |  |
|  | MEIS1B:HOXA9 | gaatacatcTGTCA | (-) | 1327 | 1 | 0,863 |  |
|  | MEIS1 | catcTGTCAcct | (-) | 1332 | 1 | 0,996 |  |
|  | Pbx-1b | tgaatCAATTaaagc | (+) | 1958 | 0,789 | 0,847 |  |
|  | Pbx-1 | gaATCAAttaaa | (+) | 1959 | 1 | 0,921 |  |
|  | Pbx-1 | atcAATTAa | (+) | 1961 | 0,931 | 0,948 |  |
|  | Pbx-1 | gctgcTTGATtt | (-) | 311 | **1** | **0,968** |  |
|  | Pbx-1 | gcgggTTGATta | (-) | 492 | 1 | 0,908 |  |
|  | Pbx-1 | tTGATTatt | (-) | 497 | **1** | **0,968** |  |
|  | MEIS1A:HOXA9 | TGAGGgtttgaaga | (+) | 544 | 0,668 | 0,798 |  |
| ma3 | MEIS1A:HOXA9 | gctgcaatcTGCCA | (-) | 635 | **0,821** | **0,757** |  |
|  | MEIS1A:HOXA9 | TTACAgctttgggg | (+) | 682 | 0,805 | 0,784 |  |
|  | Pbx-1 | tTGATTtct | (-) | 777 | 1 | 0,951 |  |
|  | Pbx-1 | tTTATTgct | (+) | 64 | 0,948 | 0,942 |  |
| 1intro1.7 | Pbx-1 | atcAATTAa | (+) | 1195 | 0,931 | 0,948 |  |
|  | MEIS1A:HOXA9 | ccatgcagcTGTTA | (+) | 1419 | 0,821 | 0,769 |  |

Distribution of binding sites from the Pbx and Meis classes. In the first column the ID of the analyzed sequence is reported. The transcription factor class and the specific consensus belonging to the class is indicated together with the matching strand and the start position of the match. Matrix and core similarity values are also indicated. Upper case in the consensus indicates the most conserved residues in the weight matrix defining the binding site.
